# Cardiovascular Risk Factors and Haematological Indexes of Inflammation in Paralympic Athletes with Different Motor Impairments

**DOI:** 10.1155/2019/6798140

**Published:** 2019-11-18

**Authors:** Marco Bernardi, Anna Lucia Fedullo, Barbara Di Giacinto, Maria Rosaria Squeo, Paola Aiello, Donatella Dante, Silvio Romano, Ludovico Magaudda, Ilaria Peluso, Maura Palmery, Antonio Spataro

**Affiliations:** ^1^Department of Physiology and Pharmacology “V. Erspamer”, Sapienza University of Rome, Italy; ^2^Comitato Italiano Paralimpico, Rome, Italy; ^3^Federazione Italiana Pallacanestro In Carrozzina (FIPIC), Rome, Italy; ^4^Institute of Sport Medicine and Science, Sport e Salute, Rome, Italy; ^5^Research Centre for Food and Nutrition, Council for Agricultural Research and Economics (CREA-AN), Rome, Italy; ^6^Department of Life, Health and Environmental Sciences, University of L'Aquila, Italy; ^7^Sport Medicine Unit AOU “G. Martino”, BIOMORF Department University of Messina, Italy

## Abstract

Haematological indexes of both inflammation and platelet activation have been suggested as predictive markers of cardiovascular disease (CVD), which has high prevalence in Paralympic athletes (PA). Different mechanisms could play a role in increasing CVD risk in PA with spinal cord injury (PA-SCI), lower limb amputation (PA-LLA), or upper limb impairment (PA-ULI). We compared, in 4 groups of PA competing in power, intermittent (mixed metabolism), and endurance sports, Framingham Risk Score (FRS), metabolic syndrome criteria (MetS-C), inflammation (INFLA) Score, 5 haematological indexes of platelet activation (mean platelet volume (MPV), platelet distribution width (PDW), and the ratios between MPV and platelet (MPVPR), between MPV and lymphocyte (MPVLR), and between PDW and lymphocyte (PDWLR)) and the endogenous antioxidants uric acid (UA) and bilirubin (BR). A retrospective chart review of PA from preparticipation examinations' records (London 2012 and Sochi 2014 Paralympics) was performed. We included 25 PA-SCI (13 with high and 12 with low lesion, PA-SCI-H and PA-SCI-L), 15 PA-LLA, and 10 PA-ULI. FRS and INFLA Score did not differ among groups, but PA-SCI-H had lower HDL, compared to PA-SCI-L and PA-ULI. PA-LLA had more MetS diagnostic criteria with significant higher glucose levels than other groups. PA-SCI-H had significantly lower lymphocytes' count compared to PA-LLA and higher MPV, PDW, MPVPR, MPVLR, and PDWLR. SCI-H had lower BR, haemoglobin, haematocrit, proteins, and creatinine. No interaction was found between the 3 kinds of sitting sports and the 2 groups of health conditions (PA-SCI and PA-LLA). In conclusion, PA-LLA had a higher cardiometabolic risk, whereas PA-SCI-H had a higher platelet-derived cardiovascular risk. Further larger studies are needed to investigate the relationship between indexes of inflammation/oxidation and dietary habit, body composition, and physical fitness/performance in PA with motor impairments.

## 1. Introduction

The common activities of daily life, carried out by individuals with spinal cord injury (SCI) or lower limb amputation (LLA), forced by their impairment to be sedentary, determine a vicious circle that perpetuates and increases sarcopenia, fat mass and osteoporosis [1], oxidative stress [2], chronic systemic inflammation [3], reduction of cardiovascular efficiency [4], dyslipidemia, insulin resistance, and atherosclerotic cardiovascular disease (CVD) risk [5]. Only physical exercise and sport are effective weapons to counter this debilitating cycle in these individuals [6], to reduce the CVD risk [1–3]. Despite the beneficial effect of exercise, high prevalence of CVD risk factors was found in Paralympic athletes with SCI (PA-SCI) and with other disorders [7]. Multiple lines of evidence have revealed common mechanisms behind cardiovascular and inflammatory diseases and clarified the metabolic and cardiovascular pathways involved in rheumatoid arthritis (RA) [8]. In particular, CVD comorbidities depend on several pathogenic mechanisms, and even if atherosclerosis is the most frequently involved, further mechanisms include microvascular dysfunction, arrhythmias, cardiac autonomic deregulation, inflammation, and immunologic abnormalities, as well as the effects of pharmacological treatments [8]. Cardiovascular autonomic nervous system dysfunction, typical in individuals with SCI, is commonly observed in RA, and it has been suggested that lowering the inflammation may represent the most effective intervention to reduce arrhythmic risk in these patients [9]. The authors suggested that these considerations could be more generally applicable to all the diseases characterized by chronic systemic inflammation [9]. Increased sympathetic activity is associated with higher mean platelet volume (MPV), with mechanisms involving peripheral activation [10]. Individuals with SCI had more extensive basal, exercise-induced [11], and oxidized-low density lipoprotein-mediated platelet activation and higher levels of lipid peroxides [12] than people without SCI. In a randomized controlled trial, a 12-week arm-cranking exercise program reduced oxidative damage and increased oxygen uptake peak in sedentary adults with SCI [2]. Regarding the antioxidant defence system, it was found that both total antioxidant status and erythrocyte glutathione peroxidase activity were significantly increased at the end of the training program, whereas plasmatic levels of lipid (malondialdehyde) and protein (carbonyl groups) oxidation markers were significantly reduced [2].

Although biomarkers of oxidative stress are relevant in the evaluation of the disease status, there is a lack of consensus concerning the validation, standardization, and reproducibility of methods for the measurement of reactive oxygen species (ROS) in leukocytes and platelets, markers based on ROS-induced modifications of lipids, DNA, and proteins, enzymatic players of redox status, and nonenzymatic antioxidant capacity of human body fluids [13]. In particular, bilirubin (BR) and/or uric acid (UA) could produce interferences in the measurement of markers of oxidative stress [13].

In a report regarding two rowers with physical impairment, qualified for the Paralympic Games in Rio 2016, high levels of BR before an exercise protocol (progressive test on a rowing ergometer until exhaustion) and during recovery (17 hours after completion of the test) were observed compared to postexercise (5 minutes postexercise), and similar trends were also observed in UA concentrations [14]. On the other hand, in individuals with chronic SCI, hyperuricemia is associated with hyperinsulinemia, elevated body mass index (BMI), and abnormal lipoprotein metabolism, but not with age or duration of injury [15]. Data from a meta-analysis reported positive relationships between UA and both nonalcoholic fatty liver disease and metabolic syndrome (MetS) [16]. In a recent retrospective chart review [17], conducted on veterans with SCI, cardiometabolic risk scores, including the most widely used CVD risk prediction Framingham Risk Score (FRS), as well as the MetS classification, may lead to different interpretations of a true risk and can account for inconsistencies between research and clinical practice. Individuals with SCI can experience blood pressure fluctuations due to neurological changes, potentially limiting the validity and/or reliability of tools used in able people in the SCI population. On the other hand, haematological indexes of inflammation/platelet activation, including platelet count (P) [18, 19], markers of platelet activation (mean platelet volume (MPV) and platelet distribution width (PDW)) [20–22], neutrophil-to-lymphocyte ratio (NLR), lymphocyte-to-monocyte ratio (LMR), platelet-to-lymphocyte ratio, mean platelet volume (MPV), and platelet distribution width (PDW) to platelet (MPVPR and PDWPR) and to lymphocyte (MPVLR and PDWLR) ratios [23, 24], have all been suggested as predictive markers of CVD risk [9, 20, 22, 25, 26], and granulocyte-to-lymphocyte ratio (GLR) has been included in the inflammation (INFLA) Score [19].

In this study, we aimed to compare the FRS, MetS criteria, INFLA Score, other haematological indexes of inflammation/platelet activation, and clinical markers (including the endogenous antioxidants UA and BR) in PA-SCI, PA-LLA, and PA with upper limb impairment (PA-ULI).

## 2. Methods

### 2.1. Selection Process of Retrospective Chart Review

We conducted a retrospective chart review of PA screened for Paralympic eligibility [27] before the Games of London 2012 and Sochi 2014. The preparticipation examinations' records, including general medical examination, instrumental, and urine and blood laboratory tests, were screened, and PA were selected based on the following criteria.

The inclusion criteria were PA-SCI, PA-LLA or PA-ULI, as well as availability of results for FRS, MetS criteria, and INFLA Score evaluations. The exclusion criteria were other health conditions (spina bifida, poliomyelitis, cerebral palsy, and other neuromuscular and/or skeletal disorders), visual impairments, and the use of anticoagulants and antiplatelet drugs. It should be stressed that platelets' indexes, among the primary outcomes of the study, could be affected (in particular) by antiplatelet drugs. Specifically, two PA were in treatment with acetylsalicylic acid and were excluded from the study, whereas none was in treatment with anticoagulants. On the other hand, the use of drugs for diabetes, hypertension, and dyslipidaemia was not considered exclusion criteria, being the use of these drugs included in the criteria for metabolic syndrome proposed by the National Cholesterol Education Program—Third Adult Treatment Panel (NCEP-ATP III).

Other characteristics of subjects, including type of impairment, sport, diseases, and use of drugs and supplements, as well as clinically relevant parameters (endogenous antioxidants, markers of anaemia, etc.), were recorded. From that, women, who can have monthly physiological variation in markers of anaemia, were also excluded. Applying the above criteria, 50 athletes were selected 25 PA-SCI, 15 PA-LLA, and 10 PA-ULI.

According to the autonomic dysfunction reported in individuals with SCI and the adrenal gland innervation [28, 29], PA-SCI were divided in two groups: 13 with high (from cervical (C)8/thoracic(T)1, incomplete, to T9, PA-SCI-H) and 12 with low (from T10 to lumbar (L)5, PA-SCI-L) lesion.

Included PA 19 practice endurance sports (3 PA-SCI-H, 6 PA-SCI-L, 2 PA-LLA, and 8 PA-ULI), 10 power sports (4 PA-SCI-H, 4 PA-LLA, and 2 PA-ULI), and 20 mixed sports (aerobic/anaerobic) (5 PA-SCI-H, 6 PA-SCI-L, and 9 PA-LLA).

### 2.2. Data Extraction and Analysis

The Framingham Score was calculated according to literature [30, 31]. MetS was evaluated by the NCEP-ATP III criteria: triglycerides (TG) ≥ 150 mg/dL or use of lipid-lowering drugs, systolic blood pressure (SBP) ≥ 130 mmHg, diastolic DBP ≥ 85 mmHg or use of antihypertensive agents, glucose ≥ 100 mg/dL or use of medications for diabetes, and in men high-density lipoprotein cholesterol (HDL) < 40 mg/dL and waist circumference > 102 cm [32]. Cholesterol/HDL and low-density lipoprotein cholesterol (LDL)/HDL ratios were also calculated [33].

INFLA Score, including C reactive protein (CRP), white blood cell (WBC), P, and GLR, was calculated as previously described [19]. Haematological indexes of inflammation and of platelet activation were calculated, including NLR, LMR, PLR, MPVPR, PDWPR, MPVLR, and PDWLR [23, 24, 26].

Other parameters were evaluated, including UA, BR, fibrinogen, proteins, creatine phosphokinase (CPK), iron, red blood cells (RBC), haemoglobin (Hb), haematocrit (HCT), mean corpuscular volume (MCV), mean cell haemoglobin (MCH), mean cell haemoglobin concentration (MCHC), the platelet/MCH ratio (PMCHR) [34], that had high values when iron-deficient anemia (IDA) was accompanied by vitamin B12 deficiency, and the serum UA to creatinine (Cr) ratio (UA/Cr), associated with a higher risk of MetS [35].

### 2.3. Statistical Analysis

Results that passed the normality test (Shapiro-Wilk) or Equal Variance Test were analyzed by analysis of variance (ANOVA), others by Kruskal-Wallis One Way Analysis of Variance on Ranks.

Two-way Analysis of Variance was conducted to evaluate the interaction between sport (endurance, power, and mixed) and impairment on data from PA-SCI (overall) and PA-LLA. PA-ULI were excluded from this analysis due to the absence of PA practicing mixed sports. The significance of the differences between groups was evaluated using the Student-Newman-Keuls or Dunn's (normality test Shapiro-Wilk and Equal Variance Test failed) methods. Spearman correlation was performed between variables (all PA, *n* = 50).

## 3. Results

### 3.1. Cardiovascular Risk

The Framingham Score did not differ among groups, but PA-SCI-H had lower HDL, compared to PA-SCI-L and PA-ULI (Table 1). Two-way ANOVA revealed that there is not a statistically significant interaction between sports and health conditions (PA-SCI and PA-LLA) (HDL *p* = 0.830).

No differences were found in CHOL, LDL, and ratios (CHOL/HDL and LDL/HDL). These ratios were correlated with both FRS (CHOL/HDL: coefficient = 0.607, *p* < 0.001, LDL/HDL: coefficient = 0.638, *p* < 0.001) and MetS ATP III (CHOL/HDL: coefficient = 0.496, *p* < 0.001, LDL/HDL: coefficient = 0.607, *p* < 0.001).

Although only 6 athletes (12%, 6/50) fulfilled the 3 criteria needed for MetS diagnosis (1 PA-SCI-H, 1 PA-SCI-L, and 4 PA-LLA), PA-LLA subjects had more criteria compared to other groups (PA-SCI-H: 1 PA with 3 criteria: W, HDL, and TG, 1 PA with 2 criteria: GLU and HDL, 5 PA with 1 criterion: 1 W, 1 BP, and 3 GLU, 6 PA no criteria; PA-SCI-L: 1 PA with 3 criteria: SBP/DBP, GLU, and TG, 1 PA with 2 criteria: GLU and BP, 3 PA with high BP only, 1 PA with low HDL only; 6 PA no criteria; PA-LLA: 1 PA with 4 criteria; BP, GLU, HDL, and TG, 3 PA with 3 criteria: 3 BP, GLU, and TG; 1 GLU, HDL, and TG, 4 PA with 2 criteria: 4 BP and GLU, 1 GLU and HDL, 5 PA with GLU only, 1 PA no criteria; PA-ULI: 7 PA with 1 criterion: 1 BP and 6 GLU, 3 PA no criteria) and GLU levels were higher in PA-LLA compared to PA-SCI (Table 1). Two-way ANOVA revealed that there is not a statistically significant interaction between sports and health conditions (PA-SCI and PA-LLA) (MetS ATP III *p* = 0.810; GLU *p* = 0.440).

### 3.2. Inflammation and Thrombotic Risk

Although means of INFLA Score, including CRP, WBC, P, and GLR, were all below 0, the value was lower in PA-ULI compared to PA-LLA (Table 2), but it did not reach a statistical significance.

PA-SCI-H had significantly lower L count compared to PA-LLA and higher MPV, PDW, and/or related platelet-activation indexes compared to other groups (Table 2). On the other hand, PA-LLA had higher M count (Table 2). The latter was correlated with CHOL/HDL (coefficient: 0.294, *p* < 0.05), TG (coefficient: 0.399, *p* < 0.01), GLU (coefficient: 0.367, *p* < 0.01), MetS (coefficient: 0.337, *p* < 0.05), N (coefficient: 0.570, *p* < 0.001), and INFLA Score (coefficient: 0.554, *p* < 0.001). Two-way ANOVA revealed that there is not a statistically significant interaction between sports and health conditions (PA-SCI and PA-LLA) for all variables (L: *p* = 0.840; M: *p* = 0.287; MPV: *p* = 0.116; PDW: *p* = 0.189; MPVPR: *p* = 0.141; MPVLR: *p* = 0.766; PDWLR: *p* = 0.702).

### 3.3. Endogenous Antioxidants, Other Clinical Parameters, and Relationship with Platelet-Activation Indexes

Among endogenous antioxidants, only BR was higher in PA-ULI compared to PA-SCI-H (Table 3), but UA/Cr was higher in PA-SCI-H and PA-SCI-L compared to PA-ULI, due to the lower levels of Cr in PA-SCI (Table 3). Moreover, compared to other groups, PA-SCI-H had lower Hb (vs. PA-LLA and PA-ULI), HCT% (vs. PA-LLA and PA-SCI-L), and proteins (vs. PA-LLA and PA-ULI) (Table 3). Two-way ANOVA revealed that there is not a statistically significant interaction between sports and health conditions (PA-SCI and PA-LLA) for variables (BR: *p* = 0.276; UA/Cr: *p* = 0.556; Cr: *p* = 0.751; Hb: *p* = 0.943; HCT: *p* = 0.984; proteins: *p* = 0.525).

Direct and inverse correlations were observed for Cr with proteins (coefficient: 0.284, *p* < 0.05), Hb (coefficient: 0.378, *p* < 0.01), iron (coefficient: 0.453, *p* < 0.01), L (coefficient: 0.415, *p* < 0.01), LMR (coefficient: 0.292, *p* < 0.05), MPVLR (coefficient: -0.351, *p* < 0.05), PDWLR (coefficient: -0.314, *p* < 0.05), and PLR (coefficient: -479, *p* < 0.001), the latter being correlated with UA/Cr (coefficient: 0.284, *p* < 0.05) and inversely related to PDW (coefficient: -0.348, *p* < 0.05) and iron (coefficient: 0.337, *p* < 0.05).

Interesting, platelet-activation indexes were inversely correlated with proteins (MPVLR coefficient: -0.349, *p* < 0.05; MPVPR coefficient: -0.323, *p* < 0.05; PDWLR coefficient: -0.299, *p* < 0.05), Cr (MPVLR coefficient: -0.351, *p* < 0.05; PDWLR coefficient: -0.314, *p* < 0.05), Hb (MPVLR coefficient: -0.528, *p* < 0.001; PDWLR coefficient: -0.509, *p* < 0.001), HCT (MPVLR coefficient: -0.459, *p* < 0.001; PDWLR coefficient: -0.459, *p* < 0.001), RBC (MPVLR coefficient: -0.405, *p* < 0.01; PDWLR -0.441, *p* < 0.01), PMCHR (MPVLR coefficient: -0.404, *p* < 0.01; MPVPR coefficient: -0.913, *p* < 0.001; PDWLR coefficient: -0.482, *p* < 0.001; PDWPR coefficient: -0.888, *p* < 0.001), GLU (MPVLR coefficient: -0.384, *p* < 0.05; MPVPR coefficient: -0.338^∗^, *p* < 0.05; PDWLR coefficient: -0.405, *p* < 0.01; PDWPR coefficient: -0.336, *p* < 0.05), MetS ATP III (MPVLR coefficient: -0.312, *p* < 0.05; MPVPR coefficient: -0.310, *p* < 0.05; PDWLR coefficient: -0.334, *p* < 0.05; PDW/P coefficient: -0.308, *p* < 0.05), and INFLA Score (MPVPR coefficient: -0.484, *p* < 0.001; PDWPR coefficient: -0.440, *p* < 0.001). On the other hand, INFLA Score was correlated with MetS (coefficient: 0.281, *p* < 0.05) and the latter was correlated with FRS (coefficient: 0.423, *p* < 0.01).

## 4. Discussion

Evidence-based screening tools designed specifically for PA to identify and classify those at CVD risk currently do not exist. In the present study, FRS did not differ among groups, whereas PA-LLA had more criteria needed for MetS diagnosis and higher GLU levels compared to both PA-SCI and PA-ULI. The complexity of identifying MetS in the population with SCI has been previously discussed, and this warrants caution in applying standard definitions of MetS to patients with SCI [17, 36]. It has been reported that variables, such as age, smoking, FRS, diabetes mellitus, CHOL, LDL, TG, and CRP, were not significantly associated with extent of coronary disease (CHD) [37]. CRP is a nonspecific marker of inflammation [38], produced predominantly in hepatocytes in response to several cytokines [39]. Although in the last decade the role of CRP level as predictor of CV events, adding prognostic information supported by the FRS, has been investigated, the improvement in CHD/CVD risk stratification or reclassification from addition of CRP to FRS was small and inconsistent [38–42]. Despite testing for CRP level is used in clinical practice, due to the fact that the test is widely available, the US Preventive Services Task Force (USPSTF) recommendation on using nontraditional risk factors in CHD risk assessment concluded that the current evidence is insufficient to assess the balance of benefits and harms of using the CRP level in risk assessment for CVD in asymptomatic adults to prevent CVD events [43]. Among the factors that constrain the predictive performance of CRP in CHD are the absence of a threshold value (general populations versus CHD patients) and the fact that CRP is not only associated with BP, CHOL, age, and gender but also with diabetes, smoking, left ventricular hypertrophy, and atrial fibrillation, all of which already contribute to the FRS [40]. Moreover, CRP levels might vary as part of the acute phase response, this limitation might bias the risk estimates toward the null and lead to an underestimation of risk [44]. Interleukin- (IL-) 6, one of the most potent drivers of CRP production, is released from activated leukocytes in response to infection or trauma and from vascular smooth muscle cells in response to atherosclerosis [39], but CRP is also released by both skeletal muscle and adipose tissue [45]. From that, body composition, sport-related energy expenditure, and physical fitness/performance parameters [46–48] could be confounding factors in the evaluation of inflammation by CRP measurement. In particular, arm-cranking exercise improved the plasma levels of inflammatory cytokines and adipokines in sedentary adults with SCI [3].

It is well known that a healthy lifestyle, including physical activity and Mediterranean diet, is essential in reducing CVD risk [41, 49]. However, in the European adolescents included in the HELENA study, despite diet has been suggested as a moderator in the association of sedentary behaviors with inflammatory biomarkers [50], the Mediterranean diet score and some healthy food subgroups were positively associated with IL-6 (pulses), alanine aminotransferase (ALT) (vegetables), and CRP (vegetables) [51]. In addition, despite monomeric CRP, stimulated by platelet activation, has prothrombotic and inflammatory properties, definitive evidence for CRP as a causative factor in atherothrombosis is lacking [39].

Probably due to all the above-mentioned confounding factors and due to the fact that the CVD risk within population subgroups may be quite different from the mean risk observed in a population [38, 43], we did not find significant differences in CRP and INFLA Score among PA-SCI-H, PA-SCI-L, PA-LLA, and PA-ULI, but INFLA Score was correlated with MetS ATP III. The latter was correlated with FRS. On the other hand, PA-SCI-H had significantly lower L count compared to PA-LLA and higher MPV, PDW, and/or related platelet-activation indexes, and inverse correlations were found for INFLA Score and MetS ATP III and platelet-activation indexes (MPVPR, MPVLR, and PDWLR).

Moreover, PA-SCI-H and/or PA-SCI-L had lower Hb (vs. PA-LLA and PA-ULI), HCT% (vs. PA-LLA and PA-SCI-L), proteins (vs. PA-LLA and PA-ULI), and creatinine (Cr) (vs. PA-LLA and PA-ULI). Inverse correlations were found for these markers, as well as BR, and platelet-activation indexes.

Although the recommendations regarding antiplatelet therapy (low-dose aspirin) in asymptomatic individuals with a moderate FRS risk are controversial [41, 43], it is known that individuals with SCI suffered a significantly higher risk of deep vein thrombosis than able people and that this risk is associated with plasma macrophage migration inhibitor [52], a regulator of innate immunity [53].

It has been reported that RBC, HGB, and HCT were negatively significantly associated with platelet aggregation, suggesting an effect of RBC-derived NO on P aggregability [54]. NO, produced in RBC membrane and cytoplasm by endothelial-type nitric oxide synthase (eNOS), inhibited platelet aggregation [54]. On the other hand, it has been reported that low concentrations of plasma amino acids, including L-arginine, the precursor for NO synthesis, in malnourished patients enhanced the occurrence of thrombotic events [55, 56]. Moreover, athletes with SCI can be at risk of low energy availability from proteins (based on the recommendation for athletes: 1.2–2.0 g of protein/kg of body weight) and of micronutrients' deficiency (in particular iron, vitamin B12, and vitamin D) [57]. In our study, no differences were found in PMCHR (marker of IDA associated with cobalamin deficiency) and mean values did not reach the cut-off value of >12.00 [35]. On the other hand, it has been reported that vitamin D deficiency may be associated with increased MPV [58], whereas malnutrition, lower values of albumin, creatinine, protein intake, and haemoglobin were associated with a higher P count [59]. In our study MPV was higher in PA-SCI-H, despite no differences in P were observed. However, it has been reported that the P of SCI group subjects did not differ significantly from those of control subjects, but platelet aggregability was higher in SCI compared to controls [11]. Besides, SCI had high platelet activation [11] and ROS-mediated damage [12]. However, in both wheelchair athletes and nonathletes, comparing to the control group, low levels of lipid oxidation (TBARS: thiobarbituric acid reactive substances) and high levels of fibrinogen have been previously reported, whereas no significant differences were found between wheelchair athletes and nonathletes in both markers [60]. Accordingly, we did not observe differences among group in fibrinogen, and within endogenous antioxidants, only BR was lower in PA-SCI-H and PA-SCI-L versus PA-ULI.

## 5. Conclusion

In this work, for the first time, we compared the FRS, the MetS criteria, the INFLA Score, and other haematological indexes of inflammation/platelet activation and clinical markers in PA-SCI-H, PA-SCI-L, PA-LLA, and PA-ULI. Despite PA-LLA had more cardiometabolic risk factors, assessed by MetS ATP III criteria, and higher GLU levels, no differences were found in FRS, INFLA Score, and CRP. PA-LLA had higher M count that was correlated with CHOL/HDL ratio, TG, GLU, MetS, and INFLA Score. On the other hand, PA-SCI-H had significantly lower L count compared to LLA and higher MPV, PDW, and/or related platelet-activation indexes (MPVPR, MPVLR, and PDWLR). Therefore, PA-LLA had a higher cardiometabolic risk, whereas PA-SCI, previously resulted a population in which the common markers of CVD risk and/or oxidative stress are not applicable [17, 36, 60], had a higher platelet-derived cardiovascular risk, probably associated with malnutrition in SCI-H. There are some limitations of our study that should be noted, including gender selection (findings may not be generalizable to women), PA-SCI subdivision and groups unbalanced for sport activity (endurance, mixed, and power) [46, 27]. Besides, inherent limitations of retrospective medical record reviews must be acknowledged, including a lack of data that could have added valuable insight such as dietary habit [41, 49]. Although further larger studies are needed to investigate the relationship among haematological indexes of inflammation and dietary habit, body composition, and physical fitness/performance [6, 61] in athletes with motor impairment, platelet-activation indexes could be among the indicators of CVD risk in SCI Paralympics.

## Figures and Tables

**Table 1:** Framingham Score and MetS criteria (NCEP-ATP III).

	PA-SCI-H (*n* = 13)	PA-SCI-L (*n* = 12)	PA-LLA (*n* = 15)	PA-ULI (*n* = 10)
Framingham score	2.3 ± 1.9	1.9 ± 1.8	3.8 ± 1.2	2.7 ± 1.2
Age (years) (sex (all men))	40.1 ± 2.0	36.6 ± 3.1	39.2 ± 1.3	37.9 ± 2.5
Smokers	2/13	1/12	4/15	1/10
CHOL (mg/dL)	178 (139-201)	207 (162-229)	209 (188-235)	200 (192-210)
HDL (mg/dL) (NCEP-ATP III: <40)	47.8 ± 2.4	63.0 ± 3.9 vs. SCI-H *p* < 0.05	54.1 ± 4.0	64.0 ± 3.7 vs. SCI-H *p* < 0.05
SBP (mmHg) (NCEP-ATP III: ≥130)	120 (110-122)	122 (120-130)	120 (110-130)	120 (117-121)
Met-S criteria (NCEP-ATP III) not included in the Framingham Score				
DBP (mmHg) (NCEP-ATP III: ≥ 85)	75 (70-80)	80 (72-85)	80 (60-85)	72 (67-80)
W (cm) (NCEP-ATP III: >102)	82 (76-98)	80 (77-90)	89 (80-97)	82 (73-87)
GLU (mg/dL) (NCEP-ATP III: ≥100)	94.9 ± 2.0 vs. LLA *p* < 0.001	96.8 ± 1.9 vs. LLA *p* < 0.001	106.9 ± 1.2	100.4 ± 1.7 vs. LLA >*p* < 0.05
TG (mg/dL) (NCEP-ATP III: ≥150)	87 (79-115)	59 (43-103)	97 (65-160)	64 (56-93)
Met-S (NCEP-ATP III)	0.5 (0.0-1.0) vs. LLA *p* < 0.05	0.5 (0.0-1.8) vs. LLA *p* < 0.05	2.0 (1.0-3.0)	1.0 (0.0-1.0)
LDL (mg/dL)	110 ± 11	124 ± 15	134 ± 9	123 ± 5
LDL/HDL	2.4 (1.5-3.2)	1.8 (1.2-2.6)	2.2 (1.8-3.8)	2.1 (1.7-2.2)
CHOL/HDL	3.7 (2.8-4.6)	3.0 (2.4-3.9)	3.4 (3.0-5.7)	3.3 (2.9-3.4)

MetS: metabolic syndrome; NCEP-ATP III: National Cholesterol Education Program—Third Adult Treatment Panel; CHOL: cholesterol; HDL: high-density lipoproteins; SBP: systolic blood pressure; DBP: diastolic blood pressure; W: waist circumference; GLU: glucose; TG: triglycerides. Data are expressed as mean and SEM (normality test (Shapiro-Wilk) passed; One Way Analysis of Variance, followed by All Pairwise Multiple Comparison Procedures (Student-Newman-Keuls Method)) or median and interquartile range (25%-75%) (normality test (Shapiro-Wilk) failed; Kruskal-Wallis One Way Analysis of Variance on Ranks, followed by All Pairwise Multiple Comparison Procedures (Dunn's Method)).

**Table 2:** INFLA Score, WBC-related, and P-related parameters.

	PA-SCI-H (*n* = 13)	PA-SCI-L (*n* = 12)	PA-LLA (*n* = 15)	PA-ULI (*n* = 10)
INFLA score	−3.3 ± 1.6	−3.6 ± 1.8	−1.3 ± 1.8	−4.6 ± 2.3
CRP (mg/L)	1.5 (0.5-4.8)	0.9 (0.4-2.9)	0.8 (0.5-1.5)	0.5 (0.2-5.0)
WBC (10^3^/microL)	5.2 ± 0.3	5.4 ± 0.4	6.4 ± 0.4	5.8 ± 0.4
P (10^3^/microL)	213.4 ± 10.9	247.2 ± 13.5	237.5 ± 13.6	220.0 ± 19.3
GLR	1.9 (1.5-2.8)	1.4 (1.2-1.9)	1.7 (1.5-1.9)	1.6 (1.1-2.0)
NLR	1.7 (1.4-2.7)	1.3 (1.1-1.9)	1.6 (1.3-1.8)	1.5 (1.1-1.9)
N (10^3^/microL)	2.9 ± 0.2	2.8 ± 0.2	3.6 ± 0.3	3.1 ± 0.3
L (10^3^/microL)	1.6 ± 0.1	1.9 ± 0.2	2.2 ± 0.1 vs. SCI-H *p* < 0.05	2.0 ± 0.1
M (10^3^/microL)	0.47 ± 0.03 vs. LLA *p* < 0.05	0.42 ± 0.03 vs. LLA *p* < 0.05	0.59 ± 0.04	0.43 ± 0.05 vs. LLA *p* < 0.05
G (10^3^/microL)	3.1 ± 0.2	3.0 ± 0.2	3.9 ± 0.3	3.3 ± 0.3
LMR	2.9 (2.5-5.4)	4.8 (3.9-5.4)	4.2 (3.1-4.3)	5.0 (4.1-6.2)
PLR	131 (100-185)	127 (114-143)	99 (97-132)	100 (96-132)
MPV (fL)	10.1 (10-10.6)	8.8 (8.7-9.5) vs. SCI-H *p* < 0.001	9.4 (8.9-9.7) vs. SCI-H *p* < 0.05	9.9 (9.6-10.1)
MPVPR (fL/10^3^/microL)	0.05 (0.04-0.06)	0.03 (0.03-0.04) vs. SCI-H *p* < 0.01	0.04 (0.03-0.05)	0.04 (0.04-0.05)
MPVLR (fL/10^3^/microL)	6.6 (5.4-8.0)	5.1 (3.7-5.6)	4.6 (3.6-5.2) vs. SCI-H *p* < 0.01	5.0 (4.4-5.5)
PDW (fL)	12.0 (11.2-14.0)	10.6 (10.0-12.1) vs. SCI-H *p* < 0.05	11.2 (10.4-12.4)	12.1 (11.4-12.7)
PDWPR (fL/10^3^/microL)	0.06 (0.05-0.08)	0.04 (0.04-0.05)	0.04 (0.04-0.06)	0.05 (0.05-0.06)
PDWLR (fL/10^3^/microL)	8.2 (6.6-9.7)	5.8 (4.4-6.7) vs. SCI-H *p* < 0.01	5.3 (4.2-6.2) vs. SCI-H *p* < 0.01	5.8 (5.5-7.1)
Fibrinogen (mg/dL)	247 (227-303)	260 (240 ± 41.4)	240 (228-270)	261 (220-299)

INFLA Score includes C reactive protein (CRP), white blood cell (WBC) and platelet (Pt) counts and granulocyte-to-lymphocyte ratio (GLR), neutrophil-to-lymphocyte ratio (NLR), neutrophil (N), lymphocyte (L), monocyte (M), granulocyte (G), lymphocyte-to-monocyte ratio (LMR), platelet-to-lymphocyte ratio (PLR), mean platelet volume to platelet (MPVPR), mean platelet volume to lymphocyte (MPVLR), platelet distribution width (PDW), platelet distribution width to platelet (PDWPR), and platelet distribution width to lymphocyte (PDWLR). Data are expressed as mean and SEM (normality test (Shapiro-Wilk) passed; One Way Analysis of Variance, followed by All Pairwise Multiple Comparison Procedures (Student-Newman-Keuls Method)) or median and interquartile range (25%-75%) (normality test (Shapiro-Wilk) failed; Kruskal-Wallis One Way Analysis of Variance on Ranks, followed by All Pairwise Multiple Comparison Procedures (Dunn's Method)).

**Table 3:** Endogenous antioxidants and other clinically relevant markers.

	PA-SCI-H (*n* = 13)	PA-SCI-L (*n* = 12)	PA-LLA (*n* = 15)	PA-ULI (*n* = 10)
UA (mg/dL)	5.6 (5.1-6.5)	6.0 (5.1-6.6)	5.8 (5.3-6.2)	5.4 (4.2-6.2)
UA/Cr	7.3 (6.5-8.2) vs. ULI *p* < 0.05	7.8 (5.7-9.1) vs. ULI *p* < 0.05	5.9 (5.8-6.8)	5.6 (4.4-5.7)
BR (mg/dL)	0.4 (0.3-0.6)	0.5 (0.5-0.7)	0.6 (0.5-0.8)	0.9 (0.6-1.4) vs. SCI-H *p* < 0.01
RBC (10^6^/microL)	4.7 ± 0.1	5.0 ± 0.1	5.0 ± 0.1	5.0 ± 0.1
Hb (g/dL)	13.6 ± 1.3	14.4 ± 0.2	14.9 ± 0.2 vs. SCI-H *p* < 0.01	14.7 ± 0.3 vs. SCI-H *p* < 0.01
HCT (%)	40.5 ± 0.8	43.0 ± 0.6 vs. SCI-H *p* < 0.05	44.2 ± 0.6 vs. SCI-H *p* < 0.01	43.1 ± 0.9
MCV (fL)	86.6 ± 1.3	85.9 ± 1.2	89.1 ± 1.3	86.6 ± 1.2
MCH (pg)	29.2 (28.4-30.5)	28.6 (27.5-30.0)	29.9 (29.3-31.4)	29.4 (28.9-29.9)
PMCHR	7.4 ± 0.5	8.5 ± 0.5	7.9 ± 0.5	7.5 ± 0.7
MCHC (g/dL)	33.7 ± 0.2	33.4 ± 0.2	33.8 ± 0.2	34.0 ± 0.2
Iron (mcg/dL)	82.7 ± 10.0	84.2 ± 8.7	106.1 ± 7.7	98.0 ± 15.0
Urea (mg/dL)	34.3 ± 2.6	41.0 ± 2.2	36.4 ± 3.1	38.9 ± 1.7
Cr (mg/dL)	0.75 ± 0.03 vs. ULI *p* < 0.001 vs. LLA *p* < 0.001	0.80 ± 0.04 vs ULI *p* < 0.01 vs. LLA *p* < 0.01	0.96 ± 0.03	1.02 ± 0.04
Proteins (g/dL)	7.0 ± 0.1	7.3 ± 0.1	7.4 ± 0.1 vs. SCI-H *p* < 0.05	7.4 ± 0.1 vs SCI-H *p* < 0.05
CPK (U/L)	161 (98-218)	197 (148-296)	142 (109-185)	152 (139-331)

UA: uric acid, BR: bilirubin, CR: creatinine, RBC: red blood cells, Hb: haemoglobin, HCT: haematocrit, MCV: mean corpuscular volume, MCH: mean cell haemoglobin, MCHPR: MCH/platelet ratio, MCHC: mean cell haemoglobin concentration, CPK: creatine phosphokinase. Data are expressed as mean and SEM (normality test (Shapiro-Wilk) passed; One Way Analysis of Variance, followed by All Pairwise Multiple Comparison Procedures (Student-Newman-Keuls Method)) or median and interquartile range (25%-75%) (normality test (Shapiro-Wilk) failed; Kruskal-Wallis One Way Analysis of Variance on, followed by All Pairwise Multiple Comparison Procedures (Dunn's Method)).

## Data Availability

The data used to support the findings of this study are restricted by the Ethics Committee in order to protect patient privacy. Data are available from Marco Bernardi and Antonio Spataro for researchers who meet the criteria for access to confidential data.
